# From fixture to function: Evaluating prosthetic complications in dental implant rehabilitation

**DOI:** 10.6026/973206300222388

**Published:** 2026-04-30

**Authors:** Harshbala Sharma, Rutvi Shah, Susmitha Murella, Ravneet Kaur, Sruthi Sakaray, Sindhu Chennamaneni

**Affiliations:** 1 Department of Dentistry, Saraswati Dental College, Lucknow, Uttar Pradesh, India; 2 Department of Prosthodontics and Crown and Bridge, KLE V. K. Institute of Dental Sciences, Belagavi, India; 3 Department of Dentistry, Government Dental College and Hospital, Vijayawada, India; 4 Department of Dentistry, Guru Nanak Dev Dental College & Research Institute, Punjab, India; 5 Department of Dentistry, Rajiv Gandhi University of Health Sciences, Karnataka, India; 6 Department of Dentistry, Panineeya Institute of Dental Sciences and Research Centre, Hyderabad, India

**Keywords:** Dental implants, prosthetic complications, implant-supported restorations, screw loosening, peri-implantitis, maintenance protocols

## Abstract

Implant-supported prostheses are widely accepted treatment modalities for replacing missing teeth in partially or completely edentulous patients. Prosthetic complications in implant dentistry can be challenging for long-term success. Insufficient hygiene care, residual cement, and poor prosthetic design are issues linked to peri-implantitis and peri-implant mucositis. Patient satisfaction is further jeopardized by aesthetic problems such as soft-tissue recession and color mismatch. Thus, we review the prevalence, factors related to complications, and the clinical impact.

## Background:

When the teeth are lost, the fundamental oromotor function, such as mastication, speech and facial esthetics, is affected, which has significant psychological ramifications for patients [1]. Full arch or partial arch implant-supported prosthetic rehabilitation is widely accepted for restoring the functions and esthetics, but this complex procedure has many challenges despite notable advancements, patient satisfaction and long-term stability [1]. Recent research reports an increasing incidence of biological, mechanical and technical complications such as framework fracture, screw loosening, ceramic chipping and marginal bone loss and peri-implant mucositis [2]. These prosthetic issues can be mitigated by preventive strategies such as prosthetic designs, controlled torque application, material selection, occlusal considerations and routine hygiene maintenance [3]. Therefore, it is of interest to report the clinical implications, influencing factors and preventive approaches for prosthetic complications in implant dentistry.

## Prosthetic complications overview:

Prosthetic complications can be broadly categorized into mechanical and biologic complications, which contribute to the failure of implant-supported crowns and bridges [4]. Poor patient selection is one of the important factors that adversely affect the success rate of Prosthetic implants [5].

## Mechanical complications:

Mechanical complications include screw loosening, abutment or screw fracture, veneer chipping off, ceramic fracture and framework breakage [6]. Overloading of components in an implant is one of the major reasons behind screw loosening or damage [7].

## Biological complications:

Bacterial infections, progressive bone loss, microbial plaque and sensory disruptions are the major consequences of biological complications. These complications are further categorized into early and late failures, where early ones are attributed to failure of placing implants during surgery and late failures are mostly peri-implantitis and bacterial plaque [8].

## Clinical impact:

Prosthetic complications can have several notable clinical consequences, as listed below:

[1] Comfort and function: Technical and mechanical complications such as chipping, framework fractures and screw loosening can compromise occlusal stability and lead to discomfort or difficulty for patients [9].

[2] Biological deterioration: The implications of biological complications are marginal and vertical bone resorption along with peri- implantitis that lead to failure of implants [10].

[3] Patient-reported outcomes: Even minor technical problems can lead to dissatisfaction, reduced quality of life, increased patient anxiety and longer chair time [11].

[4] Financial and maintenance burden: In the case of implants, patients need to be informed about the additional cost of maintenance, including time, cost and effort involved. Both technical and biological were observed at a rate of 35% over a decade of observation period.

In most cases, the restoration with implant support has good osseointegration; a considerable challenge to long-term success is noticed in prosthetic complications. In order to control and curb the occurrence of these prosthetic issues and to further improve outcomes for the patients, it is highly recommended to investigate thoroughly and apply an evidence-based approach that focuses on selected materials, methods for maintenance and designs that suit the patients [12].

## Influence of patient selection, systemic health and oral conditions on prosthetic outcomes:

Various factors can affect the effectiveness of dental implants in prosthetic rehabilitation. Choosing the suitable patient is essential. The efficacy and stability of implant-supported prostheses are greatly influenced by systemic health, patient behaviours and the oral environment. Addressing and regulating these parameters before treatment can substantially diminish the probability of biological and mechanical problems. This also enables the forecasting of treatment outcomes.

## Systemic health factors:

Systemic diseases can negatively impact osseointegration and heighten the risk of prosthetic complications. Diabetes mellitus, particularly when inadequately managed, is associated with delayed wound healing, enhanced marginal bone loss and elevated incidences of peri-implantitis [13]. Increased blood glucose levels impede the body's capacity to integrate an implant, hence reducing the probability of those with diabetes obtaining one. Likewise, smoking compromises blood circulation to tissues and reduces the effectiveness of osseointegration, hence elevating the risk of peri-implantitis. This leads to an increased incidence of prosthesis failures and diminished implant durability. Osteoporosis and the use of bisphosphonates might modify bone remodeling, potentially affecting stability and long-term integration of implants [14]. Comorbidities, including cardiovascular disease, autoimmune disorders (such as rheumatoid arthritis or lupus) and immunocompromised conditions, may hinder recovery and elevate the risk of infection. Before implant therapy, it is crucial to acquire a thorough medical history and engage with multidisciplinary clinicians to ascertain systemic factors that may affect the prosthesis outcome.

## Oral and local factors:

Oral issues significantly influence the outcome of prosthetic restorations. Residual periodontal infection, xerostomia and inadequate oral hygiene significantly contribute to peri-implant mucositis and peri-implantitis, which may result in prosthetic failure [15]. Sufficient bone and soft tissue volume is crucial for effective prosthetic results. Individuals possessing thin, non-keratinized gingiva face heightened susceptibility to recession, discomfort and aesthetic compromise. Bruxism and clenching are parafunctional habits that impose excessive stress on the occlusal components of implants. This frequently leads to screw loosening, ceramic chipping, or structural fracture [16]. Addressing plaque and inflammation, rectifying occlusion and promoting the health of soft tissues can enhance the prognosis of implant-supported prostheses by alleviating local risk factors. Before acquiring implants, it is crucial to rectify periodontally compromised or misaligned teeth, as these elements affect the prosthetic's design.

## Patient selection and maintenance:

Effective implant rehabilitation depends on careful selection and management of patients. Physicians must first evaluate both local and systemic factors to ascertain each patient's risk profile and develop appropriate treatment alternatives. A thorough oral history and a cooperative patient are essential at each stage of implant therapy to ensure lasting success. Moreover, routine maintenance appointments, standard dental cleanings and diligent home-care practices may reduce the likelihood of prosthetic complications by enabling the early identification and rectification of biological or mechanical problems. In conjunction with these clinical interventions, mitigating Parafunctional behaviours and behavioural concerns, such as smoking cessation, improves the effectiveness of the prosthesis. Implant prostheses achieve optimal efficacy through a synergistic integration of excellent clinical practices, resolution of underlying conditions and patient adherence, all guided by evidence-based risk evaluation and continuous monitoring [17].

## Mechanical and connection-related complications: framework, abutment and screw failures:

The risk of mechanical complications and failures significantly influences implant dentistry. Studies have indicated an increased rate of mechanical failures that impact implant success, such as fractures of connecting screws, coatings, implants and prostheses. According to a systematic review by Verma *et al.* the incidence rate of mechanical complications for fixed implant restorations ranged from 5.6% to 7.7%, whereas implant-supported overdentures ranged from 2.9% to 3% [4]. Goodacre *et al.* recognized 14 mechanical failures in the scientific literature, with occurrence rates ranging from 30% to 1% for overdenture clip/attachment retention loss and fixture fractures, respectively. Screw loosening was a prevalent mechanical complication linked to implant-supported prosthesis, followed by screw breakage. Furthermore, the maxillary arch exhibits a greater incidence of mechanical complications and failures attributable to reduced bone density and a thinner cortical plate in contrast to the mandibular arch [18].

## Screw loosening:

The predominant mechanical complication in implant dentistry is the loosening of abutment and prosthetic screws, which occurs in around 6% of implant prostheses. Screw loosening is proportional to the accuracy with which the implant components fit. It is often caused by factors related to non-passive castings, cantilevers, poor occlusal design, angled loads, parafunction, increased crown height space, inadequate or excessive screw torque and improper prosthesis insertion technique [19]. Since gold screws exhibit a greater elastic modulus compared to titanium, they show a reduced occurrence of screw loosening [6]. Furthermore, the internal hex design has shown reduced susceptibility to screw loosening than the external hex configuration, attributable to decreased interaction with the external component and a smaller fulcrum distance under tipping forces [20].

## Screw fracture:

Fractures of prosthetic screws account for around 4% of cases, whereas fractures of abutment screws occur in 2% of instances. The differentiation relates to the component's diameter. The primary cause of screw fracture is biomechanical stress, which results in partially unretained restorations or fatigue that correlates with the magnitude of force exerted or the frequency of cycles. Too much torque causes the screw to fracture or strip the thread components. Gold screws exhibit a greater susceptibility to fracture compared to titanium screws, due to the superior bending fracture resistance of titanium alloy [19].

## Implant fixture fracture:

Fixture fracture is a rare complication, with an incidence rate of 1% [18]. Researchers have identified two principal causes of fixture fracture: (i) mechanical overloads resulting in metal fatigue due to overload-related factors and (ii) peri-implant bone loss, which raises the likelihood of fixture fracture due to reduced bone support and impaired dissipation of occlusal forces. Studies have demonstrated that implants situated in greater masticatory stress areas like molars and premolars and smaller diameter implants like 4 mm and 3.75 mm are more prone to fracture [21].

## Abutment fracture:

The fracture of abutments is closely correlated with force factors and may result in the failure of the implant restoration. Ceramic abutments demonstrate a fracture incidence rate of 1.9-2.0%, greater than that of metallic abutments. Furthermore, no differences were observed when comparing anterior and posterior locations or internal and external configurations of ceramic abutments. Recently, hybrid titanium base-supported zirconia abutments have been proposed as a viable alternative, exhibiting fracture resistance superior to that of one-piece zirconia abutments and comparable to the custom titanium abutments [22].

## Prosthesis design, material selection and occlusal considerations in implant restorations:

Prosthesis design is very important for long-term success and prevents complications in implant-supported restorations. A well-conceived design must ensure passive fit, optimal connector dimensions and minimal cantilever extensions to reduce mechanical stress. Whether using implant-supported fixed dental prostheses (iFDPs) with pontics or splinted crowns (iSpCs), studies show that short-term clinical outcomes are generally favorable across designs, with no major differentiation [23]. Framework plays a pivotal part in supporting veneering materials and resisting functional forces. Poorly designed frameworks always cause increased stress concentrations, resulting in fractures or chipping. The shape and structure must be modified to the clinical situation and the chemical compatibility between framework and veneering materials such as matching coefficients of thermal expansion is very important to prevent tension at the interface during sintering and oral function [24].

## Material selection:

Material selection significantly influences the durability and performance of implants. Porcelain-fused-to-metal (PFM) is considered the gold standard, especially in posterior regions where occlusal forces are high. PFM frameworks offer strong resistance to fractures and clinical stability, although they require veneering for esthetics and involve labor-intensive fabrication processes [25]. The rising cost of precious alloys and the advent of digital technologies have declined PFM use [26]. Modern restorative materials such as zirconia (Zr) and lithium disilicate glass ceramics are increasingly favoured for their esthetic appeal and CAD/CAM compatibility. Monolithic ZriFDPs demonstrate superior resistance to chipping (failure rate annually < 0.46), veneered ZriFDPs with pontics show significantly higher chipping rates (4.95 annually) and reinforced glass-ceramic iFDPs exhibit higher framework fracture rates (1.0 annually), indicating limitations in their mechanical resilience [23]. Chipping of ceramic veneers remains a major complication, especially in PFM and veneered Zr restorations. Contributing factors include weak glass-ceramic properties, inadequate framework support, mismatched thermal expansion coefficients and improper sintering techniques. High occlusal forces further exacerbate these risks, underscoring the requirement for careful material pairing and precise fabrication protocols [26, 27].

## Occlusal considerations:

Occlusal overload is a major factor that is contributing to prosthetic complications in dental implants, often intensified by design flaws and material limitations. As implants do not possess a periodontal ligament, they have a low capacity to absorb occlusal stress, making them more vulnerable to mechanical failures such as screw loosening, veneer fractures and framework deformation. Improper occlusal scheme design, especially in patients with parafunctional habits like bruxism, can lead to excessive force concentration, resulting in chipping of the veneer or a fracture. Additionally, inadequate prosthesis design could not distribute occlusal loads evenly, further intensifying stress on the implant components [28]. These complications highlight the need for customizing occlusal schemes to the patient's functional dynamics and conducting regular occlusal adjustments. Such proactive measures are essential to maintain functional harmony and prevent long-term damage to implants.

## Prosthesis-induced biological complications and peri-implant tissue responses:

The long-term success of implant restorations and prostheses is determined not only by osseointegration but also by prosthetic design and maintenance. Even well-integrated implants can fail if the prosthesis introduces stress or impedes hygiene. Prosthesis-induced biological complications represent inflammatory or degenerative changes in peri-implant tissues arising from design errors, excess cement, mechanical overload, or inadequate maintenance. These complications include mucositis, peri-implantitis, soft-tissue recession and marginal bone loss [21].

## Prosthesis-induced biological complications:

Two primary mechanisms underlie these complications-microbial plaque accumulation and mechanical overload. When restorations are over- contoured or margins are placed subgingivally, plaque removal becomes difficult, encouraging anaerobic bacterial growth dominated by Porphyromonas gingivalis, Tannerella forsythia and Prevotella intermedia. The resulting inflammatory cascade involves cytokines such as interleukin-1β and tumor necrosis factor-α, which promote connective-tissue breakdown and bone resorption [29, 30]. Mechanical stress adds a second layer of damage - heavy occlusal forces or poor framework fit can produce micro-fractures in the crestal bone, ischemia and subsequent bone remodelling [8]. This interaction between infection and mechanical trauma forms the biological foundation of prosthesis-induced peri-implant disease. Multiple prosthetic variables contribute to these problems. Over-contoured crowns limit cleaning access and trap plaque, initiating mucositis [10]. Residual cement under cement-retained restorations provides a bacterial niche that provokes chronic inflammation and localized bone loss. Occlusal overload due to uneven contact distribution causes micro-movement at the bone-implant interface, resulting in marginal bone loss and a high risk of implant failure. Non-passive framework fit generates internal stress and micromotion, damaging peri-implant bone [31]. Inadequate spacing between implants (<3 mm) reduces blood flow in the inter-implant bone, leading to crater-like resorption. Deep or subgingival margins encourage anaerobic growth and hinder hygiene, while an abrupt or poorly shaped emergence profile places pressure on soft tissues, resulting in gingival recession and implant collar exposure [32].

## Peri-implant tissue responses:

Healthy peri-implant mucosa forms a biological seal that protects underlying bone from bacterial invasion. Once plaque accumulation exceeds the host's defence mechanism, inflammation develops as peri-implant mucositis, a reversible condition characterized by edema, swelling and bleeding on probing. If untreated, the inflammation progresses apically, activating osteoclasts and resulting in vertical bone loss and pocket formation. This advanced stage, peri-implantitis, appears radiographically as crater-shaped bone defects and can compromise osseointegration if not managed early. Clinically, these changes manifest as swelling, bleeding, suppuration and deepening pockets. Radiographs reveal bone loss beyond physiologic remodeling, with angular defects in overloaded regions or localized destruction around cement-retained restorations [31].

## Additional prosthesis-related complications:

Aside from microbial and biomechanical causes, various additional factors associated with prostheses may compromise peri-implant health. Inadequate torque or recurrent occlusal stress on a screw can result in screw instability and microgap formation at the abutment-implant interface, facilitating bacterial accumulation and subsequent inflammation. Fractures of abutments or frameworks due to mechanical fatigue, inadequate material selection, or improper design can disturb load distribution and inflame surrounding tissues [33]. Metal hypersensitivity or corrosion, especially from nickel alloys or poorly polished titanium, can trigger allergic reactions, mucosal erythema and cause breakdown of the peri-implant tissue [34]. Residual monomer toxicity from provisional materials, acrylic or bis-acryl resins used for temporization, can release unpolymerized monomers, irritating peri-implant soft tissues and delaying epithelial healing. Altogether, these biological complications emphasize that successful implant restoration requires not only mechanical precision but also biocompatibility, maintenance and preservation of a healthy peri-implant environment [35].

## Preventive and maintenance strategies for enhancing long-term prosthetic success:

In the context of implant dentistry, preventive and maintenance treatment for enhancing the long-term prosthetic success is focused on the delivery of individualized care, risk analysis, including periodontitis and frequent follow-up visits. Successful implants are those that remain fully functional and healthy within the oral cavity [36].

## Prevention and assessing risks:

## Patient examination:

Risk assessment of a patient is very important before introducing implants. A history of chronic periodontitis, diabetes, obesity and smoking are also some of the elements that increase complications, including peri-implantitis. Implant success is a crucial measure of the achievement of periodontal stability before treatment [37].

## Optimization of the design of the prosthesis:

The prosthesis' design is critical for reducing mechanical failures and ensuring long-term tissue health. Some important factors include optimizing the passive fit of the framework, tightening the abutment screw to a maximum preload of 10Ncm, using shorter cantilevers, using gold abutment screws, achieving optimal occlusion, selecting an internal hex connection over external hex and fabricating metal frameworks for implant-supported overdentures [38].

## Soft tissue management:

The soft tissue condition around implants is important to prevent complications. The management of soft tissue techniques can be used during or prior to the positioning of implants, particularly in areas that place high priorities on aesthetics [36].

## Oral health and maintenance:

Regular professional care: There is a need to perform scheduled check-ups of the implants that will assist in ascertaining the well- being of the tissue and the implants. The recommendations of the American College of Prosthodontists suggest that healthy implant patients ought to see the dentist at least 4 to 6 times a year.

## Sufficient plaque control:

The prime objective of the maintenance regimens is to ensure that there is proper management of the plaque to avoid the occurrence of peri-implant mucositis to peri-implantitis. Studies have suggested that patients who engage in routine maintenance therapy have a lower rate of peri-implantitis [30].

Tools that can reduce inflammation are: -

[1] Professional: Air-polishing and ultrasonic glycine powder have been proven effective in reducing the inflammation around implants.

[2] The water flossers, interdental brushes and non-abrasive toothpaste can be used for at-home care. Water flossers may be a good solution to the problem of inflammation by including chlorhexidine in the water [39].

## Removal of the Prosthesis:

The conventional approach was to periodically remove fixed prostheses to clean them. However, recent studies showed this is only required in the presence of disease or any unclean restorations [39].

## Observation and complications:

## Early detection:

Regular probing of the surrounding implants would help early identification of the existence of harmful alterations in the peri-implant region. Observing bleeding on probing (BOP) is an essential indicator of timely detection of inflammation.

## Monitoring bone levels:

Tracking marginal bone levels radiographically is an essential measure of the long-term stability and success of implants. Complications need to be detected early and dealt with effectively [37].

## Conclusion:

The likelihood of prosthetic complications and failures plays a critical role in determining the long-term success of implant therapy. Many of these adverse outcomes can be minimized through accurate diagnosis and meticulous treatment planning. Moreover, appropriate prosthesis and occlusal design, judicious material selection and adherence to a structured maintenance protocol play a critical role in ensuring durable clinical outcomes.
